# Bevacizumab loaded CalliSpheres® bronchial arterial chemoembolization combined with immunotherapy and targeted therapy for advanced lung adenocarcinoma

**DOI:** 10.3389/fphar.2023.1170344

**Published:** 2023-05-22

**Authors:** Haitao Liu, Yahua Li, Zongming Li, Xinwei Han, Kewei Ren

**Affiliations:** ^1^ Department of Interventional Radiology, The First Affiliated Hospital of Zhengzhou University, Zhengzhou, China; ^2^ Department of Radiology, Maternal and Child Health Hospital of Hubei Province, Tongji Medical College, Huazhong University of Science and Technology, Wuhan, China

**Keywords:** lung adenocarcinoma, bevacizumab, drug-eluting beads, bronchial arterial chemoembolization, immunotherapy, targeted therapy

## Abstract

**Background:** As a new drug delivery and embolization system, drug-eluted bronchial artery chemoembolization (DEB-BACE) can not only embolize the tumor blood supply artery but also load chemotherapy drugs and slowly release them into the local environment. Bevacizumab (BEV) combined with chemotherapy drugs has attained significant achievements in the first-line treatment of advanced non-squamous non-small cell lung cancer (NSCLC). The role of BEV-loaded DEB-BACE combined with immunotherapy and targeted therapy in patients with lung adenocarcinoma (LUAD) is unclear. This study was designed to evaluate the efficacy and safety of bevacizumab-loaded CalliSpheres® bronchial arterial chemoembolization combined with immunotherapy and targeted therapy in patients with lung adenocarcinoma.

**Methods:** Nine patients with LUAD who received BEV-loaded CalliSpheres® BACE combined with immunotherapy and targeted therapy from 1 Jan 2021 to Dec 2021 were included in this study. The primary endpoint was the disease control rate (DCR) and the objective response rate (ORR). The secondary endpoints were the overall survival rates (OS) at 6 months and 12 months. The tumor response was evaluated according to the mRECIST standard. Safety was assessed by the occurrences of adverse events and the severity of the adverse events.

**Results:** All patients received CalliSpheres® BACE loaded with BEV (200 mg) in combination with immunotherapy and targeted therapy. A total of nine patients received the BACE procedures 20 times, four of them received a third session of BACE, three underwent a second session of DEB-BACE, and two underwent one cycle of DEB-BACE. Partial response and stable disease were found in seven (77.8%), and two (22.2%) patients, respectively, 1 month after the last multimodal treatment. The ORR at 1, 3, 6, and 12 months was 77.8%, 66.7%, 44.4%, and 33.3%, respectively, while the DCR was 100%, 77.8%, 44.4%, and 33.3%, respectively. The OS rates at 6-and 12-month were 77.8% and 66.7%, respectively. There were no serious adverse events.

**Conclusion:** BEV-loaded CalliSpheres® transcatheter bronchial arterial chemoembolization combined with immunotherapy and targeted therapy is a promising and well-tolerated treatment for patients with lung adenocarcinoma.

## Introduction

Lung cancer (LC) is a common lung malignancy, with the highest mortality rate in the world, at 18.4%. The incidence of non-small cell lung cancer (NSCLC) accounts for more than 80% of lung cancer. Lung adenocarcinoma (LUAD), a kind of non-small cell lung cancer, accounts for approximately 50% of lung cancer (Dong et al., 2018). Surgery, chemotherapy, and radiation are the standard treatment for LUAD, but the 5-year overall survival for LUAD patients remains low and the recurrence rate is still unsatisfactory (Martin and Leighl, 2017). For patients with advanced lung cancer or weak physical condition, the above therapy may not be applicable. Immunotherapy and targeted therapy have been well-understood for the past decade. Although clinical development of immunotherapy and targeted therapy has mostly focused on monotherapy as the second line, recent advances have shifted to combination therapy as the first line. Immune checkpoint inhibitors (ICIs) are now first-line therapies for various solid and liquid tumors. Recent studies also show that ICIs can be well combined with conventional chemotherapy, so, currently, this combination is the routine treatment for most patients with metastatic NSCLC. Patients with lung adenocarcinoma might benefit from variegated palliative treatment.

Bronchial arterial chemoembolization (BACE) as an adjuvant therapy has been deemed to be an effective treatment option for NSCLC. Chemotherapy drugs can be injected into tumors by BACE and the local concentration is greatly increased with lower side effects. BACE combined with other local treatments also achieved beneficial results. Pulmonary chemoembolization is a new choice to treat lung tumors, but the best embolization, drug, and technology are still unclear. It is clear that as a new drug delivery and embolization system, DEB-BACE has a better therapeutic effect than BACE. Previous studies have confirmed the efficacy of CalliSpheres drug-eluting beads in hepatocellular carcinoma, and advanced and inoperable lung cancer (Zhou et al., 2018; Bi et al., 2022). Bevacizumab is a recombinant humanized monoclonal antibody that binds to vascular endothelial growth factor (VEGF). The mechanism is that it binds to VEGF-A, preventing its interaction with the VEGF receptor, thus resisting angiogenesis and tumor growth. BEV combined with various chemotherapy drugs can be valuable in the treatment of lung cancer, liver cancer, colorectal cancer, and ovarian cancer. BEV combined with platinum-based chemotherapy is used as the first-line treatment for patients with unresectable advanced, metastatic, or recurrent non-squamous cell NSCLC. Previous studies have shown that the use of drug-loaded microspheres loaded with chemotherapy drugs for bronchial artery infusion chemoembolization can help to improve the tumor control rate and prolong the survival time of patients (Liu et al., 2021b). According to our experiences and basic experiment, the CalliSpheres beads (CB) have a high loading efficiency of BEV; after the CB was loaded for 40 min, the loading efficiency was similar to the maximum loading efficiency. In addition, animal experiments confirmed that BEV-CB-TACE has good safety and effectiveness in the treatment of VX2 tumors. However, as far as we know, the clinical efficacy and safety of BEV-loaded DEB-BACE combined with immunotherapy and targeted therapy have not been assessed in patients with LUAD. Therefore, the present study was designed to evaluate the short-term clinical efficacy, survival profile, and safety of BEV-loaded DEB-BACE combined with immunotherapy and targeted therapy for the treatment of LUAD.

## Materials and methods

### Ethical approval

The study was approved by the Ethical Committee of the first affiliated hospital of Zhengzhou University. The ethical approval number of the study is KY201515. All methods were carried out in accordance with relevant guidelines and regulations. Written informed consent was obtained from all subjects.

### Study design and population

This was a retrospective observational study conducted on nine patients with LUAD who underwent DEB-BACE using BEV-loaded beads from 1 January 2021 to December 2021. The indications for DEB-BACE were as follows: 1) pathological diagnosis was confirmed as LUAD, 2) patients with local progression or recurrence after initial treatment or standard treatment; 3) poor candidate for or refused to receive surgical resection, radiotherapy, or chemotherapy because of severe cardiovascular disease or lung diseases; 4) expected survival time ≥3 months and no other acute diseases or active infectious diseases; 5) willingness to receive the combination therapy, etc.; and (6)Eastern Cooperative Oncology Group (ECOG) performance status≤2. The exclusion criteria were as follows: 1) patients with systemic multiple metastases; 2) without other malignancy; 3) severe liver and renal dysfunction; 4) coagulopathy or known bleeding disorders; and 5) allergic to the contrast agent.

### CalliSpheres^®^ BACE procedure

All operations were performed by the associate chief physician and chief physician. After the interventional surgery preparation, the right femoral artery was punctured under local anesthesia, and a vascular sheath (5F, Terumo Corp, Japan) was placed. The bronchial artery was selected for angiography under the guidance of preoperative enhanced CT. In addition, it was necessary to perform angiography on the thyroid trunk, internal mammary artery, intercostal artery, and diaphragmatic artery, to find out whether there were abnormal tumor blood supply branches. After defining the tumor-feeding artery, the microcatheter (Terumo Corp, Japan) was super-selected for the responsible vessel by coaxial catheter technology. Carboplatin (60–300 mg) plus pemetrexed (250–500 mg) was used for arterial infusion chemotherapy. After chemotherapy, DEE-BACE was performed. CalliSpheres beads (300–500 μm, Jiangsu Hengrui Medicine Co., Ltd., Jiangsu, China) were used to load bevacizumab (200 mg, Innovent Biologics Co., Ltd., Jiangsu, China). The drug loading method is to mix microspheres with bevacizumab at 23°C–28°C for 30 min and shake for 30 min every 5 min. Iohexol was added immediately after the loading at the volume ratio 1:1. Then, CB-BEV was slowly and carefully injected into the tumor-feeding artery manually by syringe under fluoroscopic monitoring. After embolization, angiography was performed to evaluate the embolization effect. If the tumor-feeding artery was not completely embolized, PVA particles were used for supplementary embolization. The endpoint of embolization was that the tumor-feeding artery was completely occluded or only a small amount of trunk remains. The interval was 3 weeks between sessions.

After DEB-BACE, nine patients received targeted or immunotherapy. Three patients continued to take targeted drugs and immune drugs after DEB-BACE treatment. Two patients only took targeted drugs orally and four patients took immune preparations. A total of five patients received targeted therapy. Anlotinib (8 mg or 12 mg per day, orally, Chia Tai Tianqing Pharmaceutical Group Co., Ltd., China) was used for targeted therapy. Immunological preparations included bevacizumab (200 mg every 3 weeks, intravenous infusion, Innovent Biologics Co., Ltd., China) and sintilimab (200 mg every 3 weeks, intravenous infusion, Innovent Biologics Co., Ltd., China). Targeted therapy and immunotherapy were maintained until disease progression or prohibitive toxicities.

### Evaluation of efficacy and safety

Enhanced CT of the chest was performed after therapy to evaluate its efficacy. CT images were analyzed separately by two attending radiologists with more than 3 years of experience. According to the mRECIST standard, tumor response was evaluated. The primary endpoints were the objective response rate (ORR) and the disease control rate (DCR). Tumor response was classified into four grades: complete response (CR), partial response (PR), stable disease (SD), and progressive disease (PD). The objective response rate (ORR) was defined as CR and PR, while the disease control rate (DCR) was defined as CR, PR, and SD. Local control was defined as the stopping of cancer growth at the origin or the absence of local failure. The secondary endpoints were the overall survival rates (OS) at 6 months and 12 months. Improvements in clinical indices and symptoms, adverse events, and follow-up treatments were also recorded. Adverse events and severe adverse events were graded according to the Common Terminology Criteria for Adverse Events (version 5.0).

### Follow-up

All patients received inpatient/outpatient follow-up or telephone follow-up monthly. The endpoint of follow-up was death or the end date of 1 December 2022. Follow-up contents included symptoms, blood routine examination, liver function, renal function, tumor marker, and chest CT examination. Biochemical indicators were used to evaluate the biological toxicity of drugs. CT examination was used to evaluate the efficacy of local tumors. Death was certified through relevant registrations.

### Statistical analyses

Data were described as the mean ± standard deviation, median, or count (%). Differences among groups were analyzed by Student’s t-test, χ2 test, or Wilcoxon’s rank-sum test. SPSS 26.0 (IBM Cop., Armonk, NY) was used for statistical data analysis. A *p*-value <0.05 was considered significant. Kaplan-Meier survival analysis was used to evaluate OS.

## Results

### Patient characteristics

A total of nine patients (six men and three women) were involved in this study. The ages ranged from 42 to 79 years, and the median value was 61 years old. All patients were pathologically diagnosed with adenomatous carcinoma without genetic mutation. One patient had stage II NSCLC, two patients were stage III, and six patients were stage IV. All patients successfully accepted BEV-loaded DEB-BACE. General information on patients is shown in Table 1. The detailed characteristics of each patient are shown in Table 2. All patients were treated with BEV-loaded DEB-BACE (CalliSpheres^®^ beads) combined with immunotherapy and targeted therapy. Nine patients also received transarterial infusion with carboplatin and pemetrexed. Nine patients received 20 BACE, three underwent a second session of DEB-BACE, four underwent a third session of DEB-BACE, and two underwent one cycle of DEB-BACE.

**TABLE 1:** General information of patients.

Characteristic	Value
Age (years)	61 ± 13.3 (42–79)
Height (cm)	164 ± 7.0 (155–175)
Weight (kg)	62 ± 9.6 (48–75)
Sex	
Male	6
Female	3
Symptom	
Cough and dyspnea	6
Epigastric discomfort	1
Dysphagia	1
No symptoms	1
Preoperative ECOG score	
0	4
1	4
2	1
Treatment history	
Surgical excision	2
Chest drainage	2
None	5
TNM stage	
II	1
III	2
IV	6

ECOG:performance status made by Eastern Cooperative Oncology Group.

**TABLE 2:** Patient characteristic.

Patient	Age	Sex	Symptom	Combined diseases	Lesion characteristic	Treatment	Embolized artery	Follow-up treatment	Adverse effect
1	72	Male	Cough and dyspnea	Cardiovascular disease	Left upper lobe, maximum diameter 59 mm, with obstructive pneumonia, T3N0M0, stage IIB	DEB-BACE loaded with bevacizumab (200 mg) using CB (300–500 μm), Infusion: carboplatin (300 mg), pemetrexed (500 mg)	Left bronchial artery	Targeted therapy and immunotherapy	Skin itching
						3 cycles of BAI/DEB-BACE			
2	48	Female	Cough and dyspnea	None	Right lower lobe, maximum diameter 35 mm, with obstructive pneumonia, T2N3M1, stage IVA	DEB-BACE loaded with bevacizumab (200 mg) using CB (300–500 μm), Infusion: carboplatin (300 mg), pemetrexed (500 mg)	Right intercostal artery, left bronchial artery, and right diaphragm artery	Targeted therapy	None
						2 cycles of BAI/DEB-BACE			
3	77	Male	Dysphagia	Hypertension	Light lower lobe, maximum diameter 53 mm, with obstructive pneumonia, T3N3M0, stage IIIC	DEB-BACE loaded with bevacizumab (200 mg) using CB (300–500 μm)	Right bronchial artery	Immunotherapy	None
						Infusion: carboplatin (60 mg), pemetrexed (500 mg)			
						2 cycles of BAI/DEB-BACE			
4	53	Male	Cough and dyspnea	None	Right lower lobe, 38 mm × 25 mm, T2N2M0, stage IIIA	DEB-BACE loaded with bevacizumab (200 mg) using CB (300–500 μm)	Right bronchial artery	Immunotherapy	Ankle edema
						Infusion: carboplatin (300 mg), pemetrexed (500 mg)			
						1 cycle of BAI/DEB-BACE			
5	42	Male	Epigastric discomfort	Cardiovascular disease	Left lung, 42 mm × 34 mm, with obstructive pneumonia, T2N0M1, stage IVA	DEB-BACE loaded with bevacizumab (200 mg) using CB (300–500 μm)	Left bronchial artery	Immunotherapy	None
						Infusion: carboplatin (60 mg), pemetrexed (500 mg)			
						3 cycles of BAI/DEB-BACE			
6	56	Male	Cough and dyspnea	Diabetes	Right middle lobe and left lower lobe, maximum diameter 38 mm, T3N2M1, stage IVA	DEB-BACE loaded with bevacizumab (200 mg) using CB (300–500 μm)	Branches of the left bronchial artery and left internal mammary artery	Targeted therapy	None
						Infusion: carboplatin (300 mg), pemetrexed (500 mg)			
						2 cycles of BAI/DEB-BACE			
7	79	Male	Cough and dyspnea	Hypertension	Right upper lobe and left lower lobe maximum diameter 38 mm, T2N2M1, stage IVA	DEB-BACE loaded with bevacizumab (200 mg) using CB (300–500 μm)	Bilateral bronchial arteries	Immunotherapy	None
						Infusion: carboplatin (300 mg), pemetrexed (500 mg)			
						1 cycle of BAI/DEB-BACE			
8	56	Female	Cough and dyspnea	Diabetes	Left upper lobe, 35 mm × 31 mm, T2N2M1, stage IVA	BACE: DEB-BACE loaded with bevacizumab (200 mg) using CB (300–500 μm)	Bilateral bronchial artery, left internal mammary artery	Targeted therapy and immunotherapy	Skin itching
						Infusion: carboplatin (150 mg), pemetrexed (250 mg)			
						3 cycles of BAI/DEB-BACE			
9	69	Female	No symptoms	Radical resection of lung cancer, Diabetes, Hepatitis	Left lower lobe, 15 mm × 11 mm, postoperative recurrence, with obstructive pneumonia, stage IV	DEB-BACE loaded with bevacizumab (200 mg) using CB (300–500 μm),PVA (350–560 μm)	Branches of left bronchial artery and left internal mammary artery	Targeted therapy and immunotherapy	Toothache
						Infusion: carboplatin (300 mg), pemetrexed (500 mg)			
						2 cycles of BAI/DEB-BACE			

### Efficacy

**FIGURE 1:**
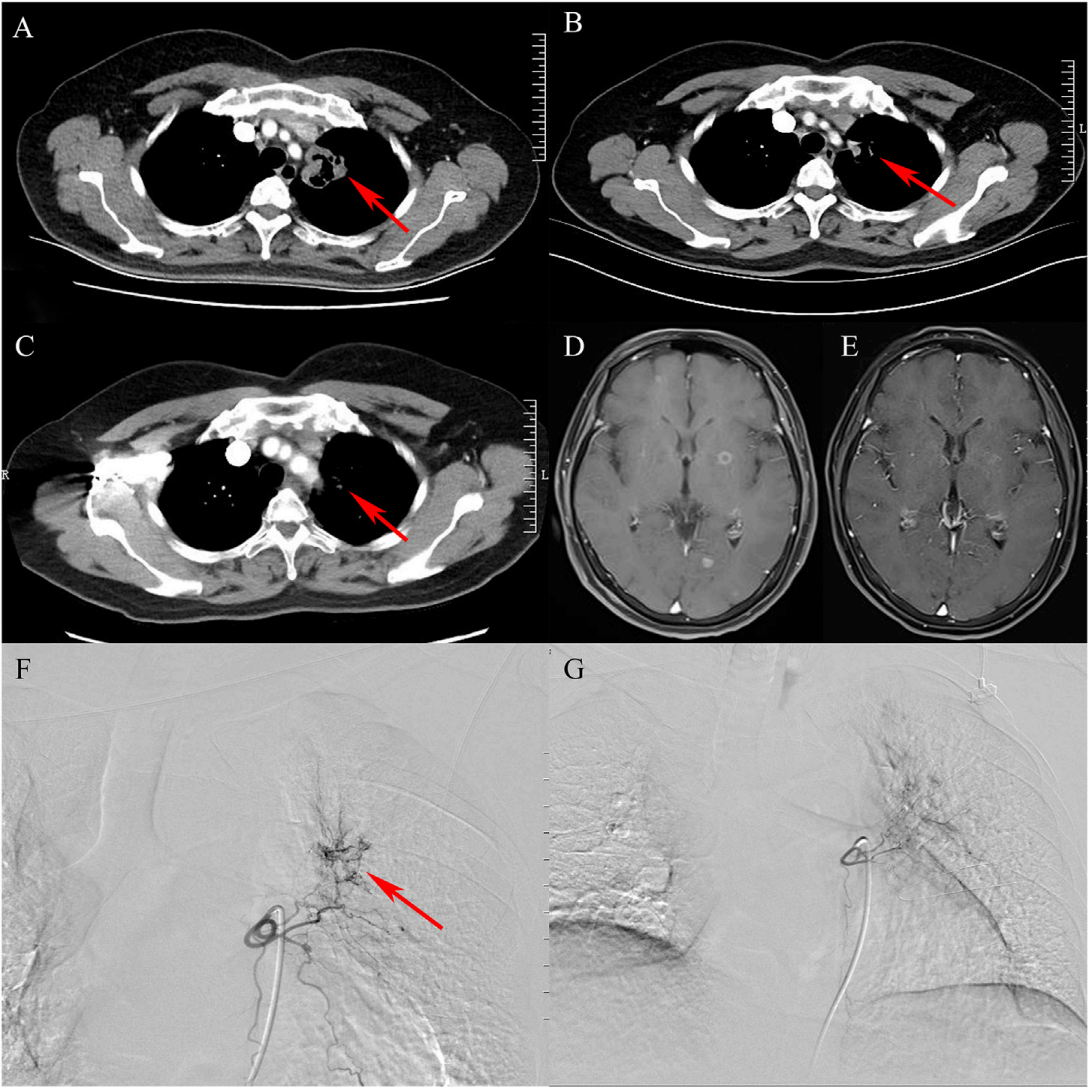
A case of lung adenocarcinoma with multiple brain metastases. **(A)**: Contrast-enhanced chest CT shows that cavitary tumor lesions are seen in the upper lobe of the left lung (arrow). **(B)**: After treatment, the tumor entity is smaller than before (arrow). **(C)**: The tumor entity is further reduced and is not enhanced (arrow). **(D)**: Multiple metastatic tumors in bilateral cerebral hemispheres (contrast-enhanced MRI, T1). **(E)**: After comprehensive treatment, the brain metastases became smaller or even disappeared (contrast-enhanced MRI, T1). **(F)**: Bronchial arteriography shows that the upper lobe of the left lung is abnormally stained and the left bronchial artery is the target artery (arrow). **(G)**: In the second cycle of treatment, the staining range of the upper lobe of the left lung is smaller than before.

**FIGURE 2:**
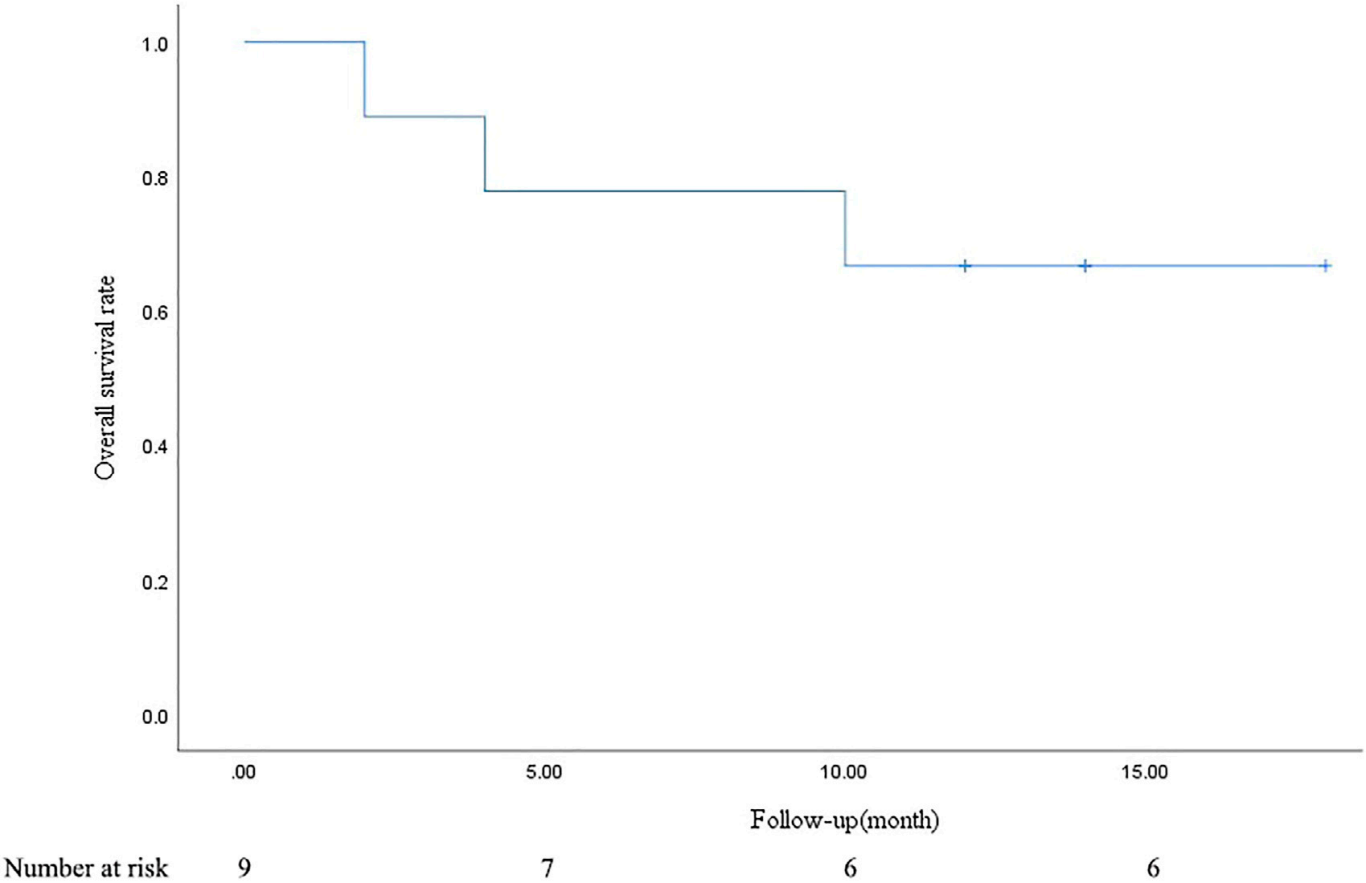
Kaplan-Meier survival analysis of overall survival rate.

PR and SD were found in seven (77.8%) and two (22.2%) patients 1 month after the combined treatment. No patient achieved CR and PD. The CR, PR, SD, and PD at 1, 3, 6, and 12 months after DEB-BACE are shown in Table 3. The ORR at 1, 3, 6, and 12 months was 77.8%, 66.7%, 44.4%, and 33.3%, respectively, while the DCR was 100%, 77.8%, 44.4%, and 33.3%, respectively. The OS rates at 6- and 12-month were 77.8% and 66.7%, respectively. In a patient with lung adenocarcinoma complicated with multiple brain metastases, after treatment, the lung lesions were significantly reduced and the brain metastases were less than before (Figure 1). Symptoms also improved significantly compared with before. Kaplan-Meier survival analysis of the overall survival rate is shown in Figure 2.

**TABLE 3:** Tumor response according to mRECIST standard (n = 9).

Response 1	1 m (%)	3 m (%)	6 m (%)	12 m (%)
CR	0 (0)	2 (22.2)	4 (44.4)	2 (22.2)
PR	7 (77.8)	4 (44.4)	0 (0)	1 (11.1)
SD	2 (22.2)	1 (11.1)	0 (0)	0 (0)
PD	0 (0)	1 (11.1)	0 (0)	0 (0)
ORR	7 (77.8)	6 (66.7)	4 (44.4)	3 (33.3)
DCR	9 (100)	7 (77.8)	4 (44.4)	3 (33.3)

CR, complete response; PR, partial response; SD, stable disease; PD, progressive disease; ORR, objective response rate; DCR, disease control rate.

### Treatment-related adverse events

Regarding safety, no serious adverse events were observed during the study period. Slight adverse reactions, such as local pain, fever, and cough, were mainly related to chemoembolization and were relieved after symptomatic treatment. This study reported four cases of adverse reactions with targeted drugs and immune agents. Two patients suffered from skin itching, which was tolerable, one patient suffered from ankle edema, which may have been related to abnormal renal function, and one patient suffered from toothache, which was relieved after oral painkillers.

## Discussion

This study shows that BEV-loaded DEB-BACE combined with immunotherapy and targeted therapy has a beneficial short-term effect on advanced lung adenocarcinoma with few complications. Transcatheter arterial embolization has achieved a beneficial effect in the treatment of solid tumors such as hepatocellular carcinoma (Galle et al., 2017; Chang et al., 2020). The principle is that chemotherapy drugs are directly injected into the tumor-feeding artery. Because of the ‘first-pass effect’, a smaller dose of chemotherapy drugs can be used to achieve a higher drug concentration in the tumor (Datta et al., 2019). Meanwhile, the toxic reaction to the whole body is greatly reduced. Previous studies have shown that pulmonary chemoembolization is a safe and effective treatment for lung, mediastinum, and bronchial metastasis (Sieghart et al., 2015). The bronchial artery is considered to be the principal tumor-feeding vessel of lung cancer. Bronchial embolization can have a beneficial effect in the treatment of lung cancers. Cao et al. found that BACE guided by DSA is effective for patients with advanced primary bronchogenic carcinoma. The short-term remission rate of 84 patients was 73.40%, and the short-term disease control rate was 93.62% (Cao et al., 2022). Drug-loaded microspheres have the functions of drug loading and drug slow release, which can keep the local drug concentration at a high level and contribute to the inhibition of tumor cells (Choi et al., 2014; Boas et al., 2021). The safety and efficacy of DEB-BACE in the treatment of advanced lung cancer have been confirmed by some studies. Yu et al. reported a case of lung adenocarcinoma, which was resected after three cycles of DEB-BACE. After the resection of the lung tumor, no tumor cells were discovered by pathology, which suggests PCR (Yu and Hu, 2020). Bie et al. found that after receiving DEB-BACE, the DCRs of NSCLC patients were 100.0%, 83.3%, and 66.7% at 2, 4, and 6 months, respectively (Bie et al., 2019). BEV combined with platinum chemotherapy is the first-line treatment for non-squamous cell carcinoma (Yang et al., 2022).

In the treatment of cancer, the combination of different modes may improve the curative effect. Different immunotherapeutic drugs, targeted therapeutic drugs, and chemotherapy drugs act on different targets and cells, which makes the synergistic or combined treatment of these drugs possible to achieve a greater therapeutic effect. However, it is worth noting that this may bring greater side effects (Yang et al., 2019). PD-1 plus CTLA-4 blockade has achieved a long-term lasting survival result over chemotherapy, but in the first few months of treatment, the survival result may be worse than chemotherapy. This may be related to the more frequent initial response of NSCLC patients to first-line chemotherapy, but it is also shorter. In order to alleviate this pattern, related randomized studies evaluated PD-1 plus CTLA-4 blocking plus chemotherapy, aiming at combining the durability of combined immunotherapy with the initial beneficial effects of chemotherapy (Reck et al., 2022). A randomized controlled study involving 719 patients showed that nivolumab plus ipilimumab plus two cycles of chemotherapy can prolong the overall survival of patients compared with the standard course of four cycles of chemotherapy (median 15.8 months *v* 11.0 months) (Paz-Ares et al., 2021). Cytotoxicity therapy can be combined with ICBs to kill tumor cells, increase the ratio of T cells to tumors, and restore metabolic restrictions that lead to low T cell reactivity of cancer. (Chang et al., 2015). In this study, the lung lesions in a patient with lung adenocarcinoma and brain metastasis were almost invisible and the brain metastases disappeared after three cycles of BEV-loaded DEB-BACE combined with immunotherapy and targeted therapy, suggesting CR.

DEB-BACE combined with different treatment methods has achieved gratifying results. To some patients with NSCLC, the combined treatment of MWA (microwave ablation) and DEB-BACE has a better local control effect than DEB-BACE, with a higher 6-month PFS rate and a longer PFS (Yang et al., 2016; Xu et al., 2022). Li et al. found that sintilimab plus DEB-BACE in NSCLC was also superior to that with monotherapy of PD-1/PD-L1 blockade as first-line therapy (Li et al., 2021). Liu et al. stated that DEB-BACE combined with anlotinib is effective in the treatment of advanced non-small cell lung cancer, and can effectively improve the OS and PFS of patients (Liu et al., 2021a). In our study, DEB-BACE loaded with BEV resulted in an ORR of 44%, while the DCR was 100% after DEB-BACE. The OS rates at 6- and 12-month were 77.8% and 66.7%, respectively. Therefore, DEB-BACE loaded with BEV is a promising and well-tolerated treatment for patients with lung adenocarcinoma.

However, it is still controversial whether DEB-BACE can prolong the progression-free survival and overall survival of LC. Many short-term studies have shown that DEB-TACE can increase the PFS rate and OS of patients. However, several long-term follow-ups show that there is no difference in survival rate between DBE-TACE and TACE in hepatocellular carcinoma (Cheung et al., 2016). Even so, DEB-BACE can be used as a palliative treatment for advanced lung cancer, effectively relieving symptoms in combination with other therapy (Chen et al., 2017). Consequently, the short-term and long-term efficacy of DEB-BACE in patients with LC needs to be confirmed by larger sample size research.

In terms of safety, DEB-BACE is similar to chemoembolization of solid tumors, mainly with post-embolization syndrome, which can be relieved after appropriate treatment. It should be noted that with DEB-BACE, there is a need to carefully observe the angiographic results to avoid ectopic embolism. The most important aspect to pay attention to is the spinal artery, with the risk of paraplegia if it is embolized by mistake. The main complications of BEV include hypertension, proteinuria, thromboembolism, and hemorrhage. In this study, two patients developed skin pruritus, which is tolerable and may be related to other oral target immune drugs. One patient suffered ankle edema that might have been related to BEV, which leads to abnormal renal function and proteinuria. Another patient developed a toothache, which was relieved after oral painkillers.

In addition, patients with lung cancer often suffer from hemoptysis. Previous reports have shown that BACE/DEB-BACE can effectively stop bleeding. Li et al. stated that BACE for advanced LC with hemoptysis is effective and tolerable, with a clinical success rate of 86.6% (Xiaobing et al., 2022). DEB-BACE combined with BEV is not suitable for lung cancer patients with hemoptysis. BEV may aggravate the bleeding. Patients with pulmonary hemorrhage/hemoptysis within 3 months should not use BEV. According to the patient’s condition, conventional chemoembolization or DEB-BACE-loaded chemotherapeutic drugs can be used. In our study, nine patients had chronic diseases of different degrees, and no serious complications occurred. Therefore, it is relatively safe to use DEB-BACE loaded with BEV for LUAD; however, more cases are needed to confirm this conclusion.

Some limitations existed in our study: First, this study is a single-center retrospective study, hence, there may be selection bias and interference factors. Second, in this study, nine LUAD patients were treated by BEV-loaded DEB-BACE combined with immunotherapy and targeted therapy retrospectively. A prospective study with larger sample size is needed to confirm the accuracy of this study. Finally, long-term follow-up is needed to evaluate the long-term efficacy.

In conclusion, BEV-loaded DEB-BACE combined with immunotherapy and targeted therapy is a promising and well-tolerated treatment for patients with lung adenocarcinoma.

## Data Availability

The raw data supporting the conclusion of this article will be made available by the authors, without undue reservation.

## Abbreviations

BEV, bevacizumab; BACE, bronchial arterial chemoembolization; CB, CalliSpheres beads; BACE, bronchial arterial chemoembolization; DEB-BACE, Drug-eluting beads bronchial arterial chemoembolization; DCR, the disease control rate; ICIs, immune checkpoint inhibitors; LUAD, lung adenocarcinoma; LC, lung cancer; MWA, microwave ablation; NSCLC, nonsquamousnon-small cell lung cancer; ORR, objective response rate; OS, overall survival; VEGF, vascular endothelial growth factor.
